# Crystal structure of 4-(1*H*-indol-3-yl)-2-(4-meth­oxy­phen­yl)-6-phenyl­pyridine-3-carbo­nitrile

**DOI:** 10.1107/S1600536814020170

**Published:** 2014-09-24

**Authors:** R. Vishnupriya, J. Suresh, Marimuthu Sakthi, Subbu Perumal, P. L. Nilantha Lakshman

**Affiliations:** a Department of Physics, The Madura College, Madurai 625 011, India; b Department of Organic Chemistry, School of Chemistry, Madurai Kamaraj University, Madurai 625 021, India; c Department of Food Science and Technology, University of Ruhuna, Mapalana, Kamburupitiya 81100, Sri Lanka

**Keywords:** crystal structure, pyridine-3-carbo­nitrile, heterocyclic compounds, hydrogen bonding

## Abstract

In the title compound, C_27_H_19_N_3_O, the dihedral angles between the plane of the pyridine ring and those of the indole (r.m.s. deviation = 0.018 Å), phenyl and meth­oxy­benzene substituents are 33.60 (6), 25.28 (7) and 49.31 (7)°, respectively. The N atom of the carbo­nitrile group is significantly displaced [0.288 (2) Å] from the plane of the pyridine ring, perhaps due to steric crowding. In the crystal, inversion dimers linked by pairs of N—H⋯N_n_ (n = nitrile) hydrogen bonds generate *R*_2_^2^(16) loops. Aromatic π–π stacking [centroid–centroid separation = 3.6906 (7) Å] and very weak C—H⋯π inter­actions are also observed".

## Related literature   

For the use of 2-amino-3-cyano­pyridines as inter­mediates in the preparation of heterocyclic compounds, see: Shishoo *et al.* (1983 ▶).
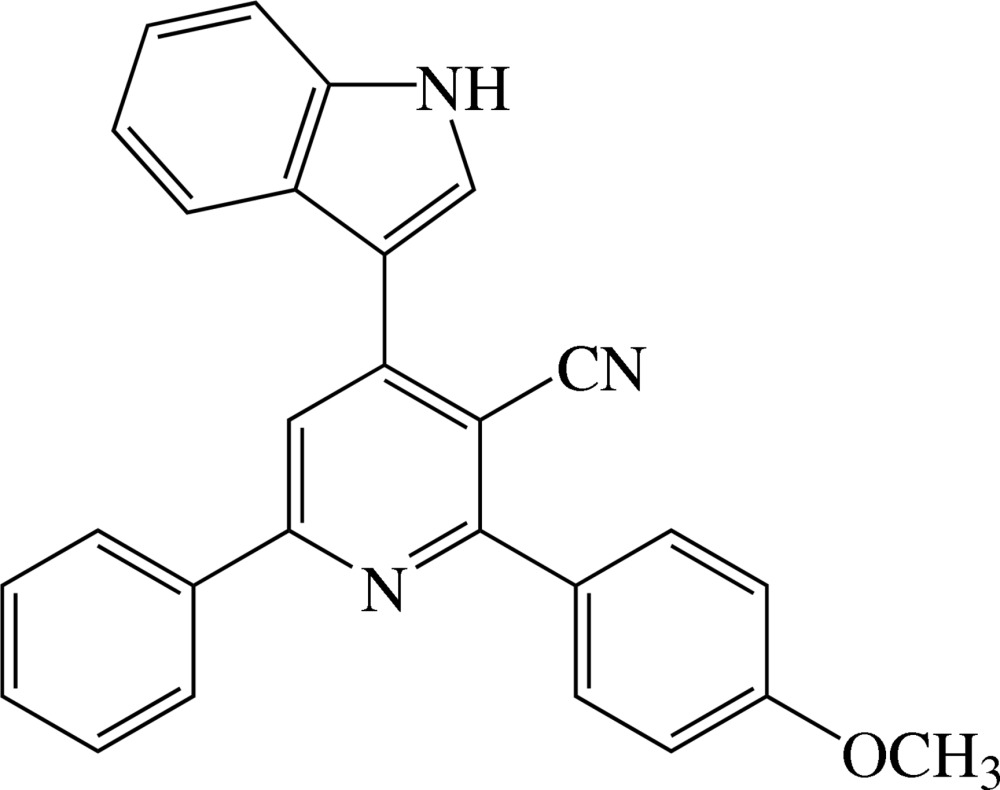


## Experimental   

### Crystal data  

C_27_H_19_N_3_O
*M* *_r_* = 401.45
Orthorhombic, 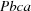

*a* = 15.7102 (5) Å
*b* = 10.7491 (3) Å
*c* = 24.3648 (7) Å
*V* = 4114.5 (2) Å^3^
*Z* = 8
Mo *K*α radiation
μ = 0.08 mm^−1^
*T* = 293 K
0.30 × 0.28 × 0.25 mm

### Data collection  

Bruker Kappa APEXII diffractometer
Absorption correction: multi-scan (*SADABS*; Sheldrick, 1996 ▶) *T*_min_ = 0.976, *T*_max_ = 0.980
27554 measured reflections
4486 independent reflections
3331 reflections with *I* > 2σ(*I*)
*R*_int_ = 0.025

### Refinement  

*R*[*F* ^2^ > 2σ(*F* ^2^)] = 0.039
*wR*(*F* ^2^) = 0.104
*S* = 1.01
4486 reflections
282 parameters
H-atom parameters constrained
Δρ_max_ = 0.15 e Å^−3^
Δρ_min_ = −0.16 e Å^−3^

### 

Data collection: *APEX2* (Bruker, 2004 ▶); cell refinement: *SAINT* (Bruker, 2004 ▶); data reduction: *SAINT*; program(s) used to solve structure: *SHELXS97* (Sheldrick, 2008 ▶); program(s) used to refine structure: *SHELXL97* (Sheldrick, 2008 ▶); molecular graphics: *PLATON* (Spek, 2009 ▶); software used to prepare material for publication: *SHELXL97*.

## Supplementary Material

Crystal structure: contains datablock(s) global, I. DOI: 10.1107/S1600536814020170/hb7280sup1.cif

Structure factors: contains datablock(s) I. DOI: 10.1107/S1600536814020170/hb7280Isup2.hkl

Supporting information file. DOI: 10.1107/S1600536814020170/hb7280Isup3.cml

**Fig. 1.**
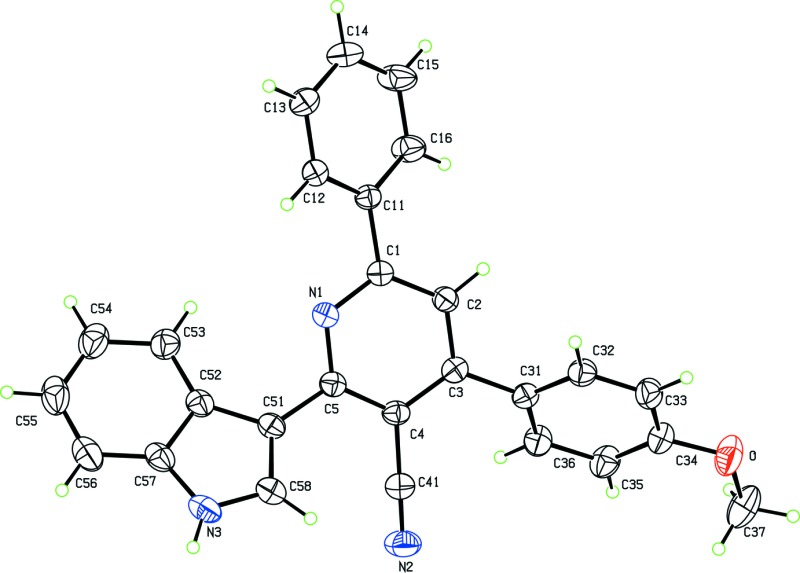
The molecular structure of compound showing 30% probability displacement ellipsoids.

**Fig. 2.**
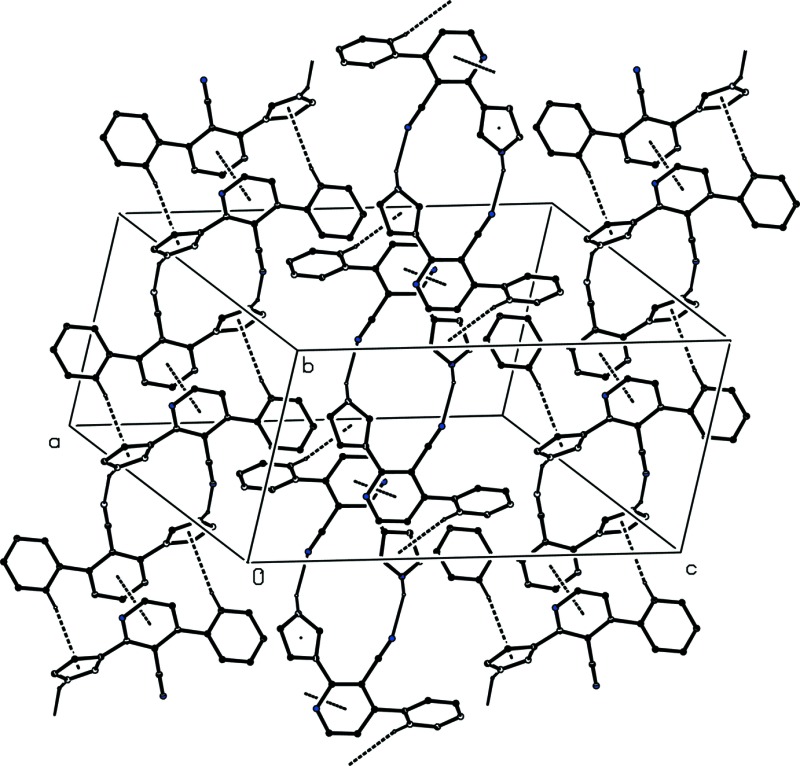
partial packing view of the compound showing molecules interconnected through a C—H···π and π···π stacking interaction (dotted lines; symmetry code: (i) (1-x, 1-y,-z)

. DOI: 10.1107/S1600536814020170/hb7280fig1.tif
The mol­ecular structure of compound showing 30% probability displacement ellipsoids.

x y z . DOI: 10.1107/S1600536814020170/hb7280fig2.tif
partial packing view of the compound showing mol­ecules inter­connected through a C—H⋯π and π⋯π stacking inter­action (dotted lines; symmetry code: (i) (1-*x*, 1-*y*,-*z*)

CCDC reference: 1023204

Additional supporting information:  crystallographic information; 3D view; checkCIF report

## Figures and Tables

**Table 1:** Hydrogen-bond geometry (Å, °) *Cg*1 is the centroid of the pyrrole ring.

*D*—H⋯*A*	*D*—H	H⋯*A*	*D*⋯*A*	*D*—H⋯*A*
N3—H3⋯N2^i^	0.86	2.15	2.9693 (19)	159
C32—H32⋯*Cg*1^ii^	0.93	3.00	3.9157 (19)	170

Symmetry codes: (i) 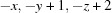; (ii) 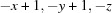.

## supplementary crystallographic information

### S1. Comment

Derivatives of 3-cyanopyridine are important and useful intermediates in preparing a varity of heterocyclic compounds (Shishoo *et al.*, 1983). Therefore, the synthesis of 3-cyanopyridine derivatives attracts much interest in organic chemistry. It was in this context that the title compound, was investigated.

The deviation of the nitrile atoms (C41,N2) from the mean plane of the pyridine ring system is -0.1497 (1) Å and -0.2886 (5) Å. The shortening of the C—N distances [1.337 (3) and 1.341 Å] and the opening of the N1–C11–C10 angle [121.15 (2)°] may be attributed to the size of the substituent at C1, correlating well with the values observed in the *ortho*-substituted derivative. The dihedral angle between the pseudo-axial phenyl substituent and the plane of the pyridine ring is 69.13 (8)°.

The crystal structure features an N—H···N interaction between inverse related molecules generating a graph set ring motif *R*_2_^2^ (16) which are linked into chains through C—H···*Cg*1 interation (*Cg*1 is the centroid of the pyrrole ring of the indole moiety) and by π···π stacking interaction involving adjacent pyridine rings of the symmetry related molecule at (1-*X*,1-Y,-*Z*), with a centroid-to-centroid distance of 3.6906 (7) Å·(Fig 2).

### S2. Experimental

A mixture of 3-(1*H*-indol-3-yl)-3-oxopropanenitrile 1 (1 mmol), 4,4,4-trifluoro-1- phenylbutane-1,3-dione 2 (1 mmol) and 4-methoxy benzaldehyde 3 (1 mmol) in the presence of ammonium acetate (400 mmol) under solvent-free condition was heated at 110°C for 7 h. After completion of the reaction (TLC), the reaction mixture was poured into water and extracted with dichloromethane. After removal of the solvent, the residue was chromatographed over σilica gel (230–400 mesh) using petroleum ether-ethyl acetate mixture (7:3 *v*/*v*), which afforded the pure compound.

Melting point:265 °C, Yield: 72%.

### S3. Refinement

H atoms were placed at calculated positions and allowed to ride on their carrier atoms with C—H = 0.93–0.98 Å and with *U*_iso_ = 1.2*U*_eq_(C, N) for N, CH_2_ and CH atoms and *U*_iso_ = 1.5*U*_eq_(C) for CH_3_ atoms.

### Crystal data

**Table:**

C_27_H_19_N_3_O	*F*(000) = 1680
*M**_r_* = 401.45	*D*_x_ = 1.296 Mg m^−^^3^
Orthorhombic, *P**b**c**a*	Mo *K*α radiation, λ = 0.71073 Å
Hall symbol: -P 2ac 2ab	Cell parameters from 2000 reflections
*a* = 15.7102 (5) Å	θ = 2–27°
*b* = 10.7491 (3) Å	µ = 0.08 mm^−^^1^
*c* = 24.3648 (7) Å	*T* = 293 K
*V* = 4114.5 (2) Å^3^	Block, colourless
*Z* = 8	0.30 × 0.28 × 0.25 mm

### Data collection

**Table:**

Bruker Kappa APEXII diffractometer	4486 independent reflections
Radiation source: fine-focus sealed tube	3331 reflections with *I* > 2σ(*I*)
Graphite monochromator	*R*_int_ = 0.025
Detector resolution: 0 pixels mm^-1^	θ_max_ = 27.0°, θ_min_ = 2.1°
ω and φ scans	*h* = −20→20
Absorption correction: multi-scan (*SADABS*; Sheldrick, 1996)	*k* = −13→8
*T*_min_ = 0.976, *T*_max_ = 0.980	*l* = −31→31
27554 measured reflections	

### Refinement

**Table:**

Refinement on *F*^2^	Secondary atom site location: difference Fourier map
Least-squares matrix: full	Hydrogen site location: inferred from neighbouring sites
*R*[*F*^2^ > 2σ(*F*^2^)] = 0.039	H-atom parameters constrained
*wR*(*F*^2^) = 0.104	*w* = 1/[σ^2^(*F*_o_^2^) + (0.0411*P*)^2^ + 1.0149*P*] where *P* = (*F*_o_^2^ + 2*F*_c_^2^)/3
*S* = 1.01	(Δ/σ)_max_ = 0.001
4486 reflections	Δρ_max_ = 0.15 e Å^−^^3^
282 parameters	Δρ_min_ = −0.16 e Å^−^^3^
0 restraints	Extinction correction: *SHELXL97* (Sheldrick, 2008), Fc^*^=kFc[1+0.001xFc^2^λ^3^/sin(2θ)]^-1/4^
Primary atom site location: structure-invariant direct methods	Extinction coefficient: 0.0027 (4)

### Special details

**Table:**

Geometry. All e.s.d.'s (except the e.s.d. in the dihedral angle between two l.s. planes) are estimated using the full covariance matrix. The cell e.s.d.'s are taken into account individually in the estimation of e.s.d.'s in distances, angles and torsion angles; correlations between e.s.d.'s in cell parameters are only used when they are defined by crystal symmetry. An approximate (isotropic) treatment of cell e.s.d.'s is used for estimating e.s.d.'s involving l.s. planes.
Refinement. Refinement of *F*^2^ against ALL reflections. The weighted *R*-factor *wR* and goodness of fit *S* are based on *F*^2^, conventional *R*-factors *R* are based on *F*, with *F* set to zero for negative *F*^2^. The threshold expression of *F*^2^ > σ(*F*^2^) is used only for calculating *R*-factors(gt) *etc*. and is not relevant to the choice of reflections for refinement. *R*-factors based on *F*^2^ are statistically about twice as large as those based on *F*, and *R*- factors based on ALL data will be even larger.

### Fractional atomic coordinates and isotropic or equivalent isotropic displacement parameters (Å^2^)

**Table:**

	*x*	*y*	*z*	*U*_iso_*/*U*_eq_	
C1	0.11716 (8)	1.07304 (12)	0.96916 (5)	0.0400 (3)	
C2	0.14114 (9)	1.06339 (12)	1.02373 (5)	0.0436 (3)	
H2	0.1574	1.1344	1.0428	0.052*	
C3	0.14124 (8)	0.94978 (12)	1.05007 (5)	0.0416 (3)	
C4	0.11465 (8)	0.84683 (11)	1.01907 (5)	0.0416 (3)	
C5	0.08413 (8)	0.86400 (11)	0.96531 (5)	0.0407 (3)	
C11	0.12231 (8)	1.19255 (12)	0.93922 (5)	0.0402 (3)	
C12	0.07016 (9)	1.21553 (12)	0.89470 (5)	0.0465 (3)	
H12	0.0316	1.1552	0.8833	0.056*	
C13	0.07492 (11)	1.32728 (14)	0.86716 (6)	0.0559 (4)	
H13	0.0391	1.3420	0.8375	0.067*	
C14	0.13180 (11)	1.41679 (14)	0.88303 (7)	0.0605 (4)	
H14	0.1350	1.4917	0.8641	0.073*	
C15	0.18372 (11)	1.39515 (15)	0.92690 (8)	0.0711 (5)	
H15	0.2223	1.4558	0.9380	0.085*	
C16	0.17937 (10)	1.28396 (14)	0.95483 (7)	0.0614 (4)	
H16	0.2152	1.2701	0.9846	0.074*	
C31	0.16647 (9)	0.94381 (12)	1.10856 (5)	0.0430 (3)	
C32	0.13218 (10)	1.03013 (13)	1.14465 (6)	0.0515 (4)	
H32	0.0935	1.0886	1.1317	0.062*	
C33	0.15425 (11)	1.03077 (14)	1.19904 (6)	0.0576 (4)	
H33	0.1301	1.0888	1.2227	0.069*	
C34	0.21220 (10)	0.94553 (14)	1.21888 (6)	0.0547 (4)	
C35	0.24701 (10)	0.85934 (15)	1.18375 (6)	0.0586 (4)	
H35	0.2862	0.8015	1.1968	0.070*	
C36	0.22377 (10)	0.85875 (14)	1.12909 (6)	0.0532 (4)	
H36	0.2473	0.7997	1.1057	0.064*	
C37	0.28200 (13)	0.8619 (2)	1.29741 (7)	0.0858 (6)	
H37A	0.2574	0.7814	1.2910	0.129*	
H37B	0.2861	0.8763	1.3362	0.129*	
H37C	0.3378	0.8651	1.2814	0.129*	
C41	0.11876 (9)	0.72404 (13)	1.04200 (6)	0.0492 (3)	
C51	0.04591 (9)	0.76538 (12)	0.93237 (6)	0.0441 (3)	
C52	0.04502 (9)	0.75788 (12)	0.87349 (6)	0.0462 (3)	
C53	0.08391 (10)	0.82422 (15)	0.83125 (6)	0.0572 (4)	
H53	0.1182	0.8925	0.8390	0.069*	
C54	0.07081 (13)	0.78724 (17)	0.77795 (7)	0.0721 (5)	
H54	0.0967	0.8311	0.7496	0.087*	
C55	0.01956 (14)	0.68547 (18)	0.76559 (8)	0.0784 (6)	
H55	0.0120	0.6623	0.7291	0.094*	
C56	−0.01989 (12)	0.61900 (15)	0.80601 (8)	0.0704 (5)	
H56	−0.0543	0.5511	0.7977	0.085*	
C57	−0.00692 (10)	0.65614 (13)	0.85977 (7)	0.0533 (4)	
C58	−0.00419 (10)	0.66969 (13)	0.95069 (6)	0.0529 (4)	
H58	−0.0145	0.6513	0.9874	0.063*	
N1	0.08729 (7)	0.97572 (9)	0.94093 (4)	0.0419 (3)	
N2	0.12503 (10)	0.62657 (12)	1.05973 (6)	0.0694 (4)	
N3	−0.03648 (9)	0.60601 (11)	0.90767 (6)	0.0597 (4)	
H3	−0.0704	0.5435	0.9101	0.072*	
O	0.22989 (9)	0.95450 (12)	1.27328 (4)	0.0808 (4)	

### Atomic displacement parameters (Å^2^)

**Table:**

	*U*^11^	*U*^22^	*U*^33^	*U*^12^	*U*^13^	*U*^23^
C1	0.0366 (7)	0.0401 (7)	0.0433 (7)	0.0005 (5)	0.0008 (5)	0.0017 (5)
C2	0.0461 (8)	0.0390 (7)	0.0457 (7)	−0.0014 (6)	−0.0031 (6)	−0.0012 (6)
C3	0.0385 (7)	0.0436 (7)	0.0427 (7)	0.0036 (5)	0.0014 (5)	0.0027 (6)
C4	0.0398 (7)	0.0374 (7)	0.0477 (7)	0.0027 (5)	0.0046 (6)	0.0030 (6)
C5	0.0383 (7)	0.0384 (7)	0.0453 (7)	0.0021 (5)	0.0043 (6)	−0.0007 (6)
C11	0.0390 (7)	0.0396 (7)	0.0420 (7)	0.0000 (5)	0.0025 (5)	0.0025 (5)
C12	0.0513 (8)	0.0437 (7)	0.0444 (7)	−0.0006 (6)	−0.0023 (6)	−0.0008 (6)
C13	0.0626 (10)	0.0533 (8)	0.0518 (9)	0.0050 (7)	−0.0070 (7)	0.0100 (7)
C14	0.0608 (10)	0.0472 (8)	0.0736 (11)	0.0003 (7)	0.0047 (8)	0.0200 (8)
C15	0.0608 (10)	0.0561 (9)	0.0963 (13)	−0.0219 (8)	−0.0149 (9)	0.0197 (9)
C16	0.0568 (9)	0.0567 (9)	0.0706 (10)	−0.0151 (7)	−0.0196 (8)	0.0164 (8)
C31	0.0436 (7)	0.0424 (7)	0.0429 (7)	0.0017 (6)	0.0001 (6)	0.0037 (6)
C32	0.0586 (9)	0.0455 (8)	0.0504 (8)	0.0109 (7)	−0.0023 (7)	0.0032 (6)
C33	0.0707 (10)	0.0544 (9)	0.0477 (8)	0.0103 (8)	0.0017 (7)	−0.0055 (7)
C34	0.0593 (9)	0.0642 (9)	0.0407 (7)	0.0013 (8)	−0.0038 (7)	0.0020 (7)
C35	0.0568 (9)	0.0660 (10)	0.0528 (8)	0.0164 (7)	−0.0072 (7)	0.0068 (7)
C36	0.0550 (9)	0.0555 (8)	0.0490 (8)	0.0152 (7)	−0.0012 (7)	−0.0019 (7)
C37	0.0742 (12)	0.1313 (17)	0.0517 (10)	0.0134 (12)	−0.0137 (9)	0.0174 (11)
C41	0.0482 (8)	0.0456 (8)	0.0539 (8)	0.0005 (6)	0.0004 (6)	0.0042 (6)
C51	0.0444 (7)	0.0367 (7)	0.0511 (8)	0.0035 (6)	−0.0004 (6)	−0.0023 (6)
C52	0.0466 (7)	0.0389 (7)	0.0531 (8)	0.0107 (6)	−0.0068 (6)	−0.0024 (6)
C53	0.0616 (10)	0.0562 (9)	0.0537 (9)	0.0091 (7)	−0.0037 (7)	0.0016 (7)
C54	0.0878 (13)	0.0767 (12)	0.0519 (9)	0.0216 (10)	−0.0066 (9)	0.0040 (9)
C55	0.1021 (15)	0.0748 (12)	0.0582 (11)	0.0315 (11)	−0.0277 (10)	−0.0137 (9)
C56	0.0829 (13)	0.0511 (9)	0.0773 (12)	0.0176 (9)	−0.0367 (10)	−0.0141 (9)
C57	0.0554 (9)	0.0389 (7)	0.0655 (10)	0.0110 (6)	−0.0160 (7)	−0.0060 (7)
C58	0.0548 (9)	0.0423 (7)	0.0616 (9)	−0.0015 (6)	0.0010 (7)	−0.0032 (7)
N1	0.0427 (6)	0.0382 (6)	0.0447 (6)	−0.0008 (5)	0.0002 (5)	−0.0003 (5)
N2	0.0780 (10)	0.0480 (8)	0.0823 (10)	−0.0025 (7)	−0.0103 (8)	0.0158 (7)
N3	0.0606 (8)	0.0397 (6)	0.0787 (10)	−0.0065 (6)	−0.0109 (7)	−0.0045 (6)
O	0.0949 (10)	0.1018 (10)	0.0457 (6)	0.0184 (8)	−0.0140 (6)	−0.0034 (6)

### Geometric parameters (Å, º)

**Table:**

C1—N1	1.3370 (16)	C33—H33	0.9300
C1—C2	1.3857 (18)	C34—O	1.3577 (17)
C1—C11	1.4795 (17)	C34—C35	1.375 (2)
C2—C3	1.3796 (18)	C35—C36	1.381 (2)
C2—H2	0.9300	C35—H35	0.9300
C3—C4	1.4035 (18)	C36—H36	0.9300
C3—C31	1.4806 (18)	C37—O	1.417 (2)
C4—C5	1.4069 (18)	C37—H37A	0.9600
C4—C41	1.4347 (18)	C37—H37B	0.9600
C5—N1	1.3406 (16)	C37—H37C	0.9600
C5—C51	1.4590 (18)	C41—N2	1.1375 (17)
C11—C12	1.3817 (18)	C51—C58	1.3700 (19)
C11—C16	1.3833 (19)	C51—C52	1.4368 (19)
C12—C13	1.3780 (19)	C52—C53	1.393 (2)
C12—H12	0.9300	C52—C57	1.405 (2)
C13—C14	1.369 (2)	C53—C54	1.374 (2)
C13—H13	0.9300	C53—H53	0.9300
C14—C15	1.365 (2)	C54—C55	1.391 (3)
C14—H14	0.9300	C54—H54	0.9300
C15—C16	1.377 (2)	C55—C56	1.365 (3)
C15—H15	0.9300	C55—H55	0.9300
C16—H16	0.9300	C56—C57	1.384 (2)
C31—C36	1.3771 (19)	C56—H56	0.9300
C31—C32	1.3872 (19)	C57—N3	1.367 (2)
C32—C33	1.370 (2)	C58—N3	1.3507 (19)
C32—H32	0.9300	C58—H58	0.9300
C33—C34	1.379 (2)	N3—H3	0.8600
			
N1—C1—C2	122.03 (12)	C35—C34—C33	119.47 (13)
N1—C1—C11	116.42 (11)	C34—C35—C36	119.89 (14)
C2—C1—C11	121.55 (12)	C34—C35—H35	120.1
C3—C2—C1	120.85 (12)	C36—C35—H35	120.1
C3—C2—H2	119.6	C31—C36—C35	121.33 (14)
C1—C2—H2	119.6	C31—C36—H36	119.3
C2—C3—C4	116.57 (12)	C35—C36—H36	119.3
C2—C3—C31	119.10 (12)	O—C37—H37A	109.5
C4—C3—C31	124.31 (11)	O—C37—H37B	109.5
C3—C4—C5	119.94 (11)	H37A—C37—H37B	109.5
C3—C4—C41	120.15 (12)	O—C37—H37C	109.5
C5—C4—C41	119.91 (12)	H37A—C37—H37C	109.5
N1—C5—C4	121.15 (12)	H37B—C37—H37C	109.5
N1—C5—C51	114.99 (12)	N2—C41—C4	177.55 (17)
C4—C5—C51	123.85 (12)	C58—C51—C52	106.12 (12)
C12—C11—C16	118.24 (12)	C58—C51—C5	127.06 (13)
C12—C11—C1	120.66 (12)	C52—C51—C5	126.44 (12)
C16—C11—C1	121.11 (12)	C53—C52—C57	118.51 (14)
C13—C12—C11	120.40 (13)	C53—C52—C51	134.80 (14)
C13—C12—H12	119.8	C57—C52—C51	106.67 (13)
C11—C12—H12	119.8	C54—C53—C52	118.99 (16)
C14—C13—C12	120.70 (14)	C54—C53—H53	120.5
C14—C13—H13	119.6	C52—C53—H53	120.5
C12—C13—H13	119.6	C53—C54—C55	121.26 (18)
C15—C14—C13	119.45 (14)	C53—C54—H54	119.4
C15—C14—H14	120.3	C55—C54—H54	119.4
C13—C14—H14	120.3	C56—C55—C54	121.20 (16)
C14—C15—C16	120.36 (15)	C56—C55—H55	119.4
C14—C15—H15	119.8	C54—C55—H55	119.4
C16—C15—H15	119.8	C55—C56—C57	117.69 (17)
C15—C16—C11	120.85 (14)	C55—C56—H56	121.2
C15—C16—H16	119.6	C57—C56—H56	121.2
C11—C16—H16	119.6	N3—C57—C56	130.11 (16)
C36—C31—C32	117.89 (13)	N3—C57—C52	107.52 (13)
C36—C31—C3	123.59 (12)	C56—C57—C52	122.35 (16)
C32—C31—C3	118.50 (12)	N3—C58—C51	110.08 (14)
C33—C32—C31	121.22 (13)	N3—C58—H58	125.0
C33—C32—H32	119.4	C51—C58—H58	125.0
C31—C32—H32	119.4	C1—N1—C5	119.08 (11)
C32—C33—C34	120.19 (14)	C58—N3—C57	109.59 (13)
C32—C33—H33	119.9	C58—N3—H3	125.2
C34—C33—H33	119.9	C57—N3—H3	125.2
O—C34—C35	125.05 (14)	C34—O—C37	118.26 (14)
O—C34—C33	115.48 (14)		
			
N1—C1—C2—C3	5.0 (2)	C33—C34—C35—C36	0.0 (3)
C11—C1—C2—C3	−175.75 (12)	C32—C31—C36—C35	−0.4 (2)
C1—C2—C3—C4	−1.04 (19)	C3—C31—C36—C35	178.13 (14)
C1—C2—C3—C31	−179.38 (12)	C34—C35—C36—C31	0.5 (3)
C2—C3—C4—C5	−4.40 (19)	N1—C5—C51—C58	−143.80 (14)
C31—C3—C4—C5	173.84 (12)	C4—C5—C51—C58	35.2 (2)
C2—C3—C4—C41	175.41 (13)	N1—C5—C51—C52	28.20 (19)
C31—C3—C4—C41	−6.3 (2)	C4—C5—C51—C52	−152.85 (13)
C3—C4—C5—N1	6.38 (19)	C58—C51—C52—C53	−178.06 (16)
C41—C4—C5—N1	−173.43 (12)	C5—C51—C52—C53	8.6 (3)
C3—C4—C5—C51	−172.52 (12)	C58—C51—C52—C57	0.18 (15)
C41—C4—C5—C51	7.7 (2)	C5—C51—C52—C57	−173.18 (13)
N1—C1—C11—C12	24.87 (18)	C57—C52—C53—C54	−0.6 (2)
C2—C1—C11—C12	−154.39 (13)	C51—C52—C53—C54	177.47 (15)
N1—C1—C11—C16	−154.96 (14)	C52—C53—C54—C55	0.1 (2)
C2—C1—C11—C16	25.8 (2)	C53—C54—C55—C56	0.3 (3)
C16—C11—C12—C13	−0.5 (2)	C54—C55—C56—C57	−0.2 (3)
C1—C11—C12—C13	179.65 (13)	C55—C56—C57—N3	−178.46 (16)
C11—C12—C13—C14	0.6 (2)	C55—C56—C57—C52	−0.3 (2)
C12—C13—C14—C15	−0.6 (3)	C53—C52—C57—N3	179.24 (13)
C13—C14—C15—C16	0.4 (3)	C51—C52—C57—N3	0.66 (15)
C14—C15—C16—C11	−0.3 (3)	C53—C52—C57—C56	0.7 (2)
C12—C11—C16—C15	0.3 (2)	C51—C52—C57—C56	−177.84 (14)
C1—C11—C16—C15	−179.83 (15)	C52—C51—C58—N3	−0.98 (16)
C2—C3—C31—C36	−132.13 (15)	C5—C51—C58—N3	172.33 (13)
C4—C3—C31—C36	49.7 (2)	C2—C1—N1—C5	−3.18 (19)
C2—C3—C31—C32	46.38 (19)	C11—C1—N1—C5	177.56 (11)
C4—C3—C31—C32	−131.82 (14)	C4—C5—N1—C1	−2.49 (18)
C36—C31—C32—C33	−0.2 (2)	C51—C5—N1—C1	176.49 (11)
C3—C31—C32—C33	−178.80 (14)	C51—C58—N3—C57	1.44 (17)
C31—C32—C33—C34	0.7 (2)	C56—C57—N3—C58	177.06 (16)
C32—C33—C34—O	179.74 (15)	C52—C57—N3—C58	−1.28 (16)
C32—C33—C34—C35	−0.6 (3)	C35—C34—O—C37	−6.1 (3)
O—C34—C35—C36	179.65 (16)	C33—C34—O—C37	173.55 (16)

### Hydrogen-bond geometry (Å, º)

Cg1 is the centroid of the pyrrole ring.

**Table:**

*D*—H···*A*	*D*—H	H···*A*	*D*···*A*	*D*—H···*A*
N3—H3···N2^i^	0.86	2.15	2.9693 (19)	159
C32—H32···*Cg*1^ii^	0.93	3.00	3.9157 (19)	170

Symmetry codes: (i) −*x*, −*y*+1, −*z*+2; (ii) −*x*+1, −*y*+1, −*z*.
