# Supplementary figures and images for: Crystal structure of 4-(1H-indol-3-yl)-2-(4-meth­oxy­phen­yl)-6-phenyl­pyridine-3-carbo­nitrile
Source: Acta Crystallogr Sect E Struct Rep Online. 2014 Sep 24;70(Pt 10):o1120–1. doi: 10.1107/S1600536814020170 (PMC4257225; doi:10.1107/S1600536814020170)

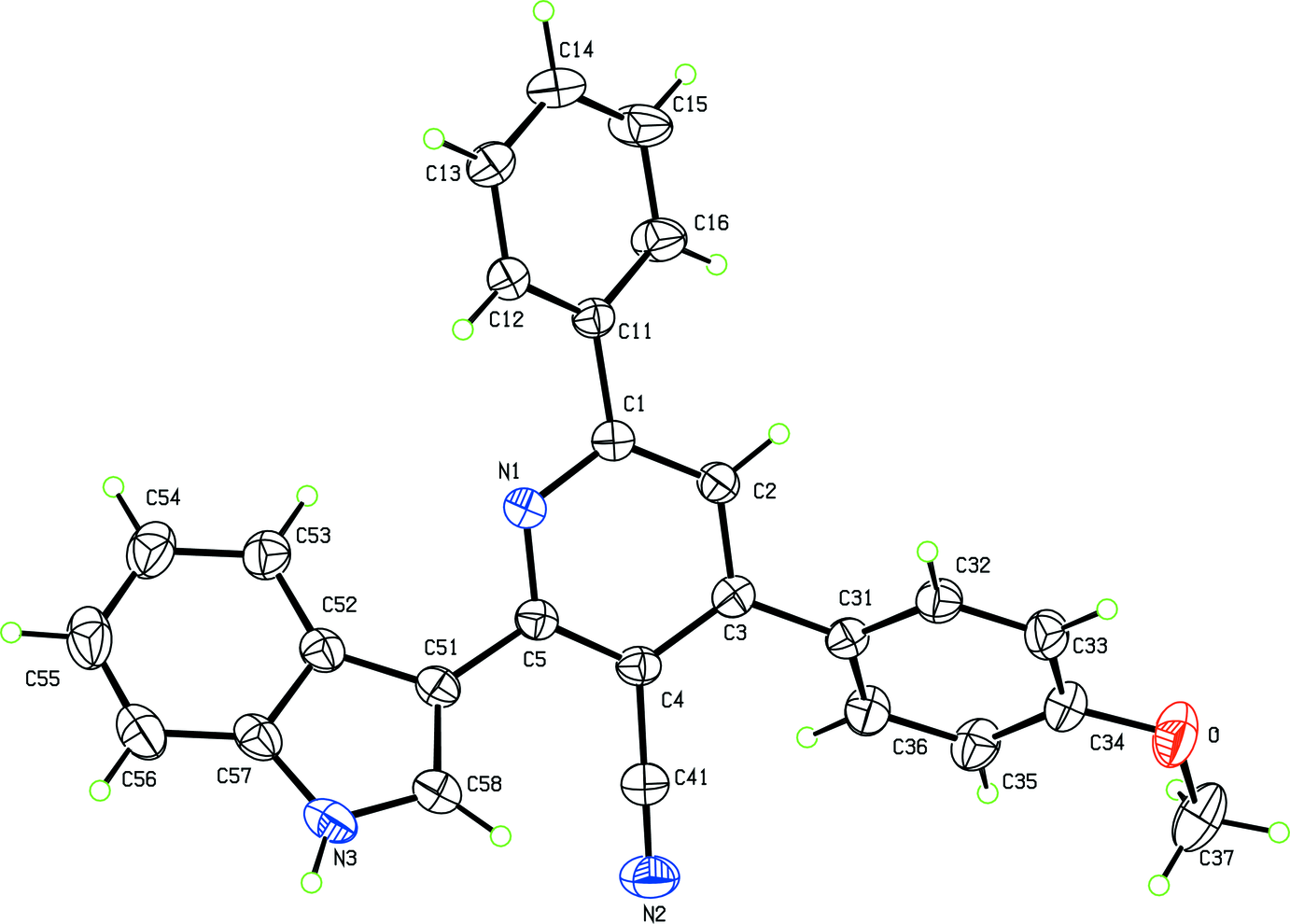

Supplement: Supplementary file 4 [file e-70-o1120-fig1.tif]

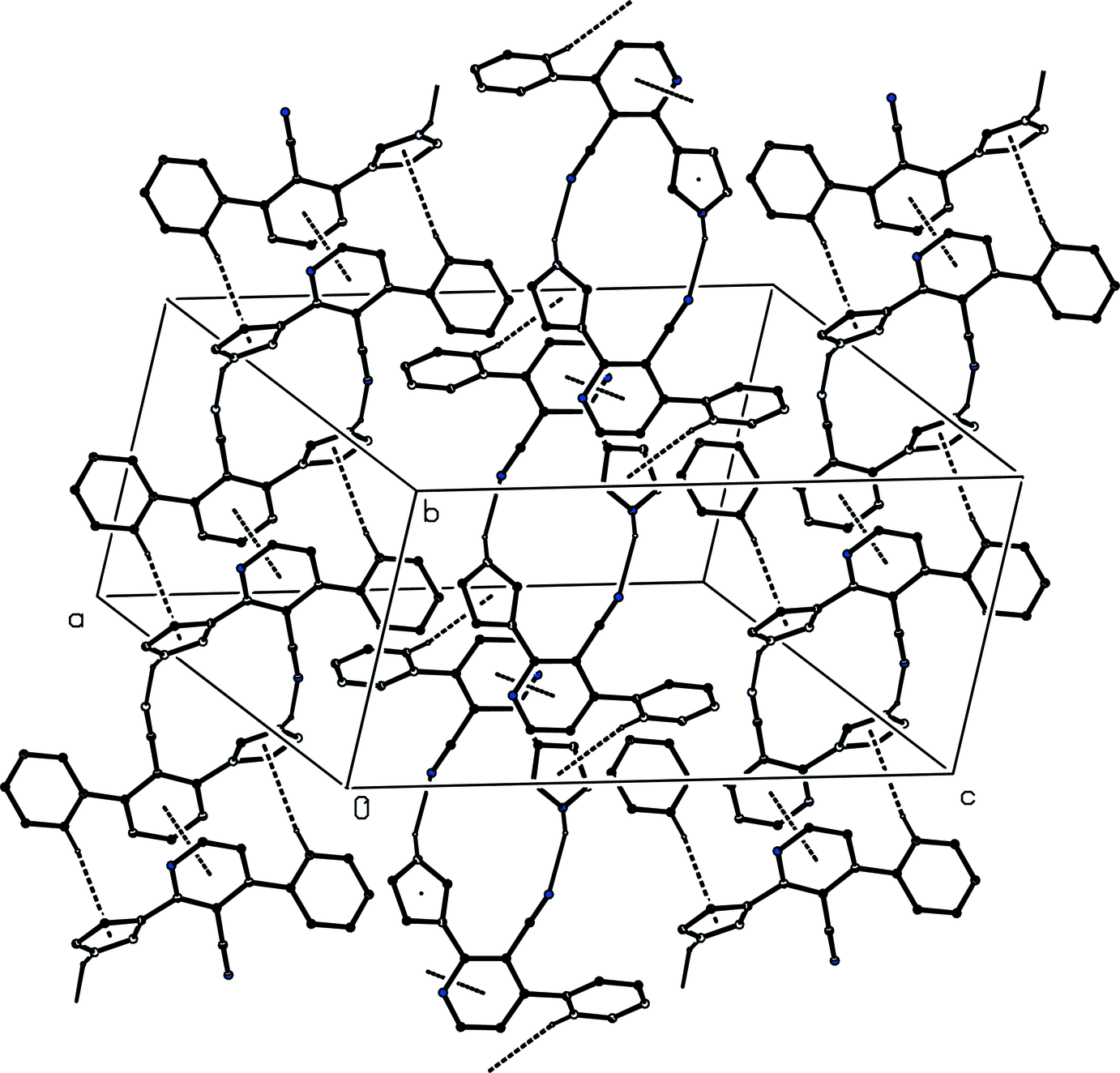

Supplement: Supplementary file 5 [file e-70-o1120-fig2.tif]
